# Long‐Term Oxygen Therapy and Cognitive Function in Chronic Obstructive Pulmonary Disease: A Systematic Review

**DOI:** 10.1155/pm/5525054

**Published:** 2026-05-14

**Authors:** Shaikh Fawwad, Saed S. K. Makhlouf, Mazen Mahgoub, Roba Sabri Kamel Makhlouf, Sumaiya Monjur, Nandita Thapar

**Affiliations:** ^1^ Department of Medicine, University of Toledo, Toledo, Ohio, USA, utoledo.edu; ^2^ Department of Medicine, Huazhong University of Science and Technology, Wuhan, Hubei, China, hust.edu.cn; ^3^ Department of Medicine, Alexandria University, Alexandria, Egypt, alexu.edu.eg; ^4^ Department of Medicine, Al-Quds University, Jerusalem Governorate, State of Palestine, alquds.edu; ^5^ Department of Medicine, Dhaka Medical College and Hospital, Dhaka, Bangladesh, dmc.edu.bd; ^6^ Department of Internal Medicine, Manipal College of Medical Sciences, Kaski, Nepal, manipal.edu.np

## Abstract

**Objective:**

This study is aimed at evaluating the association between long‐term oxygen therapy (LTOT) and cognitive function in patients with chronic obstructive pulmonary disease (COPD).

**Methods:**

We conducted a systematic literature search across major databases for observational studies comparing cognitive outcomes between COPD patients receiving LTOT (≥ 15 h/day) and those not receiving it. Cognitive performance was evaluated using validated tools like the Mini‐Mental State Examination (MMSE), Montreal Cognitive Assessment (MoCA), and Trail Making Tests (TMT‐A/B). Due to heterogeneity in study design and outcome measures, a narrative synthesis was performed.

**Results:**

We included five studies involving 1849 participants (343 LTOT users) in this review. LTOT use was generally associated with a lower prevalence of cognitive impairment (18%–45%) and higher global cognitive scores, particularly in executive functions. MoCA‐based assessments also consistently favored LTOT users, particularly in rural populations. However, findings were heterogeneous across studies.

**Conclusion:**

LTOT is associated with better cognitive performance in hypoxemic COPD patients. Prospective longitudinal studies are needed to establish causality between LTOT use and reduction in cognitive impairment in COPD.

## 1. Introduction

Chronic obstructive pulmonary disease (COPD) is a chronic, irreversible, progressive respiratory disorder characterized by airflow limitation, chronic hypoxemia and hypercapnia, and systemic inflammation [1, 2]. Apart from its primary pulmonary manifestations, COPD has been recognized as a multisystemic entity causing cardiovascular dysfunction, skeletal muscle weakness, sarcopenia, and cognitive impairment [3, 4]. Cognitive impairment in COPD is a concerning comorbidity, with a prevalence ranging from 20% to 30%. It affects multiple domains, including memory, attention, executive function, and psychomotor speed [5, 6]. This complicates disease management, adherence to therapy, and severely affects the overall quality of life of the patients [7].

Cognitive impairment in COPD has been associated with the risk factors such as aging, smoking, low partial pressure of oxygen in blood (PaO_2_), and socioeconomic factors such as low educational level [5]. The underlying pathophysiology linking COPD to cognitive impairment is multifactorial. Chronic hypoxemia, hypercapnia, systemic inflammation, and cerebrovascular events are some of the contributing factors of cognitive decline. However, chronic hypoxia seems to be the predominant factor for the cognitive decline in COPD [8]. Chronic hypoxemia reduces cerebral oxygen delivery, leading to neuronal injury and degeneration in different areas of the brain, particularly in the hippocampus and cortical areas, resulting in cognitive disturbances. Multiple neuroimaging studies have demonstrated that lower arterial oxygen saturation (SaO_2_) and PaO_2_ are associated with greater white matter lesions, hippocampal atrophy, and deficits in executive function and memory [9, 10].

Long‐term oxygen therapy (LTOT) is the cornerstone intervention for patients with severe hypoxemia in COPD. Current guidelines from the Global Initiative for Chronic Obstructive Lung Diseases (GOLD) and the American Thoracic Society recommend LTOT for adults with COPD who have severe resting hypoxemia, defined as a PaO_2_ ≤ 55 mmHg (7.3 kPa) or SaO_2_ ≤ 88% on room air, or PaO_2_ of 56–59 mmHg (7.5–7.9 kPa) with features of right heart failure, polycythemia, or pulmonary hypertension [11, 12]. Even though LTOT is conventionally prescribed to improve survival and reduce hospitalizations, it may also play a role in preserving cognitive function by correcting hypoxemia and reducing hypoxia‐induced neuronal injury [9, 13]. Furthermore, LTOT has been reported to improve cerebral blood flow velocity, autonomic function, and neuropsychological performance, particularly in domains sensitive to oxygenation such as psychomotor speed and executive function [7]. Likewise, LTOT can alleviate dyspnea and improve exercise tolerance, allowing participation in physical and social activities that are considered protective against cognitive decline in COPD [14].

Multiple prior reviews have described the association between hypoxia and cognitive impairment in COPD [5, 6]. However, these studies have primarily focused on the burden and risk factors of cognitive impairment in COPD, with insufficient discussion on the therapeutic effects of LTOT on hypoxemic COPD patients. LTOT can significantly increase oxygen delivery to brain regions involved in cognitive function through mechanisms related to cerebral blood flow. In patients with COPD, cerebrovascular insensitivity impairs the normal cerebral vasoconstriction response to elevated blood oxygen saturation. As a result, LTOT is able to raise the total oxygen content in the blood without causing a reduction in cerebral blood flow. Moreover, LTOT raises the peak conductance of cerebral vessels during neural activity by up to 40%. This reduces the reliance on high systemic blood pressure to maintain cerebral perfusion [13]. Despite being known as the possible intervention option for cognitive impairment in COPD, the evidence regarding its efficacy remains limited and inconsistent. Given these discrepancies and the biological plausibility that LTOT may ameliorate cognitive deficits in COPD, a synthesis of the available evidence is required. This systematic review is aimed at evaluating the effect of LTOT on cognitive function in COPD patients.

## 2. Methods

### 2.1. Registration of Research

We conducted the systematic review using the Preferred Reporting Items for Systematic Reviews and Meta‐Analyses (PRISMA) 2020 guidelines and criteria [15]. We registered the review on the PROSPERO database (CRD420261321765, https://www.crd.york.ac.uk/PROSPERO/view/CRD420261321765). We also utilized the AMSTAR‐2 checklist to assess the quality of our review [16]. The overall quality of our review was rated as moderate. The full PRISMA checklist of the study is available in the supporting information.

### 2.2. Search Strategy

We performed a literature search across major databases including PubMed, Embase, Scopus, and Web of Science from inception to December 2025 using a comprehensive search strategy (Table S1). The last search was performed on January 10, 2026 to ensure the inclusion of the most recent literature. We used the keywords and MeSH headings related to COPD, LTOT, and cognitive impairment along with the Boolean operators to create a search strategy.

We screened the reference lists of included studies and other relevant reviews on the topic to identify additional eligible articles. Additionally, we contacted a few authors via e‐mail and ResearchGate to obtain full texts and clarify the dilemmas wherever required.

### 2.3. Eligibility Criteria

We included observational or interventional studies in patients 40 years and above with COPD that reported the effect of LTOT (≥ 15 h/day or prescribed as continuous home oxygen) on cognitive function assessed using validated screening tools (Mini‐Mental State Examination (MMSE), Montreal Cognitive Assessment (MoCA), and neuropsychological batteries).

Studies involving non‐COPD populations (e.g., asthma and interstitial lung disease), studies reporting the effect of acute oxygen therapy and nonoxygen therapy‐based interventions, studies without cognitive outcomes, studies on pediatric populations, non‐English studies, nonpeer‐reviewed studies, case reports, editorials, or narrative reviews were excluded from this systematic review.

### 2.4. Study Selection

Two reviewers independently screened titles and abstracts for relevant articles. We retrieved the full texts of potentially eligible studies and assessed them against the inclusion criteria. We resolved any disagreements between the reviewers through a discussion under the mediation of a third reviewer. The study selection process was documented using a PRISMA flow diagram, detailing the number of records identified, screened, excluded, and included.

### 2.5. Data Extraction

We extracted data into a standardized form in MS Excel 2019 on the study characteristics (author, publication year, country, and design), patient demographics, sample size, LTOT duration, cognitive assessment tools used, and reported outcomes.

### 2.6. Quality Assessment

The methodological quality of included studies was appraised using the Newcastle–Ottawa Scale (NOS) for observational studies. Domains assessed included selection of participants, comparability of groups, and outcome assessment. Studies were categorized as low, moderate, or high risk of bias [17]. All authors participated in the quality assessment of the included studies.

### 2.7. Data Synthesis

Given the heterogeneity in study designs, cognitive measures, and LTOT protocols, a narrative synthesis was performed. We assessed heterogeneity qualitatively by systematically comparing studies across three domains: (1) clinical characteristics (COPD severity/baseline oxygen saturation), (2) intervention parameters (LTOT duration and reported adherence), and (3) methodological factors (cognitive assessment tools used). Where possible, we reported the effect sizes (e.g., odds ratios, relative risk, and mean differences), but meta‐analysis was not feasible due to methodological variability.

## 3. Results

### 3.1. Study Selection

**Figure 1:**
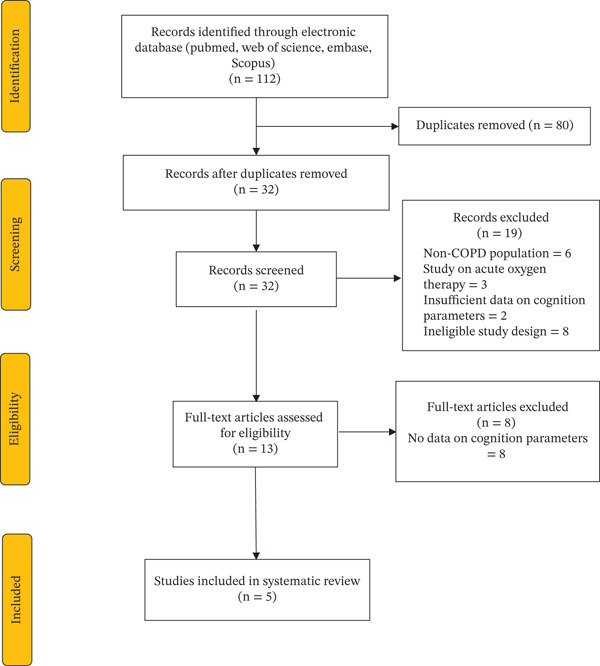
PRISMA flow diagram of selection of studies.

The literature search yielded a total of 112 articles from the database. After the exclusion of duplicates and studies that did not meet the eligibility criteria, a total of five studies were included in this review. Figure 1 shows the results of our literature search and selection.

### 3.2. Study Characteristics

Out of five studies, four were cross‐sectional comparison studies [18–21], and one was a large cohort study [8]. The sample size of the studies ranged from 45 to 1202. The total sample size was 1849. The total number of participants receiving LTOT was 343, and the number of nonusers was 1506.

All five studies compared the cognition level between LTOT users and LTOT nonusers using various validated cognitive assessment tools. Global cognitive performance was assessed using validated tools, most commonly the MMSE and/or MoCA. Four studies used the MMSE and two studies used the MoCA to quantify the cognitive impairment in the participants. One study additionally compared the executive function measures between the two groups using the Trail Making Test A/B (TMT‐A/B). We avoided meta‐analysis due to heterogeneity in LTOT definition, variable severity of COPD among the study population, and lack of enough published studies with larger populations. Hence, a narrative synthesis of findings structured by cognitive domain/tool was performed in this review. We have provided the details on the included studies in Table 1.

**Table 1:** Characteristics of included studies.

SN	Study	Study site	Design	COPD severity	Sample size (users/nonusers)	Mean age of participants (years)	Duration of LTOT	Cognition assessment tools	Adherence to LTOT
1	Karamanli 2015	Turkey	Cross‐sectional	Severe hypoxemia (FEV1 < 30%) with no exacerbation, PaO2 ≤ 55 mmHg	45 (21/24)	69.3 ± 10	24 h/day	MMSE MoCA	Not mentioned
2	Cilingir 2020	Turkey	Cross‐sectional	Hypoxemic COPD patients (mean FEV1% = 53.8 ± 9.4, mean PaO2 = 53.7 ± 8.6)	84 (22/62)	62.3 ± 10.2	24 h/day	MMSE	Not mentioned
3	Chen 2023	China	Cross‐sectional	Hypoxemic COPD patients (FEV1% < 48.87%)	372 (166/206)	69.37 ± 9.12	≥ 15 h/day	MoCA (Beijing version)	63 participants (16.9%)
5	Negro 2015	Italy	Cross‐sectional	Hypoxemic COPD patients (mean FEV1% = 41.6 ± 11.1, mean PaO2 = 54.2 ± 8.3)	146 (73/73)	70.5 ± 12.9	≥ 15 h/day	MMSE TMA A, B	Not mentioned
6	Thakur 2010	United States	Cohort study (FLOW study)	Hypoxemic COPD patients (mean FEV1% = 52.0 ± 21.0)	1202 (61, 1141)	58.2 ± 6.2	≥ 15 h/day	MMSE	Not mentioned

Abbreviations: FEV1%, FEV1/FVC (forced expiratory volume in 1 s/Forced vital capacity); MMSE, Mini‐Mental State Examination; MoCA, Montreal Cognitive Assessment.

The quality of the studies included in the review was variable, ranging from fair to very high. Two studies [18, 19] were rated fair, two [20, 21] were rated moderate, and one [8] of the studies was rated very high quality level (Table S2).

### 3.3. Global Cognitive Performance (MMSE and MoCA)

#### 3.3.1. MMSE

A total of four studies [8, 18–20] used MMSE scores to compare the cognitive impairment between LTOT users and nonusers. The impact of LTOT on the cognition of stable COPD patients as measured by MMSE scores was variable across studies.

Participants with MMSE ≤ 24 were considered to have cognitive impairment. Across these studies, the prevalence of MMSE‐defined cognitive impairment among LTOT users ranged from approximately 18% to 45%, compared with 35% to over 70% among non‐LTOT users [18, 21].

Two out of four studies reported on the quantitative benefit of LTOT. Karamanli et al. [18] reported significantly higher MMSE scores in LTOT users compared with nonusers: 25.89 ± 2.79 versus 23.75 ± 2.63, respectively (*p* = 0.014). Likewise, in the FLOW cohort [8], regular home oxygen use was associated with a marked reduction in the MMSE‐defined cognitive impairment (adjusted odds ratio (aOR): 0.14, 95% CI 0.07–0.27, *p* < 0.0001).

In contrast, Çilingir et al. [19] observed lower MMSE scores in LTOT users who received regular continuous oxygen therapy (18.81 ± 3.65) compared with nonusers (24.9 ± 5.17). Furthermore, Dal Negro et al. [20] found no statistically significant difference in MMSE score among LTOT users (> 15 h/day) and those using intermittent oxygen “as needed” (20.1 ± 3.1 vs. 22.0 ± 2.3, *p* = 0.34).

#### 3.3.2. MoCA

Two studies [18, 21] used the MoCA tool to compare the cognitive impairment between users and nonusers of LTOT with more consistent quantitative findings favoring LTOT. Participants with MoCA < 26 were considered to have cognitive impairment.

The prevalence of MoCA‐defined cognitive impairment was observed lower among LTOT users (36.8%–44.6%) compared with LTOT nonusers (66.0%–70.8%) [18, 21]. Karamanli et al. [18] observed a significantly better performance in MoCA with regular use of LTOT: 21.68 ± 2.14 in regular LTOT users versus 19.37 ± 2.99 in nonusers (*p* = 0.007). Likewise, Chen et al. [21] reported a significant protective association between LTOT and MoCA‐defined cognitive impairment in rural COPD patients (aOR: 0.133, 95% CI: 0.041–0.436). However, the same benefit could not be observed in urban participants (aOR: 0.804, 95% CI: 0.221–2.922).

### 3.4. Executive Function Outcomes

Only Dal Negro et al. [20] compared executive function using TMT‐A and TMT‐B between LTOT users and LTOT nonusers. LTOT use for more than 15 h per day was associated with better performance on both tests. LTOT users performed significantly better in both TMT‐A test and TMA‐B test as compared with the nonusers (TMT‐A: 132.2 ± 35.8 vs. 155.3 ± 52.5, *p* = 0.012) (TMT‐B: 322.1 ± 36.2 vs. 344.2 ± 31.8, *p* = 0.001) which showed better processing speed and executive performance.

### 3.5. Summary of Findings

The effect of LTOT on global cognitive performance and executive function outcomes is variable among the studies. The direction of effect of LTOT on cognitive outcomes across the included studies is summarized in Table 2.

**Table 2:** Summary of findings across included studies.

Study	MMSE	MoCA	Executive function	Overall direction
Thakur 2010	Favoring LTOT: significantly higher scores in users with 86% reduction in impairment risk for users.	Not assessed	Not assessed	Favoring LTOT
Karamanli 2015	Favoring LTOT: significantly higher scores in users (25.9 vs. 23.8).	Favoring LTOT: consistently higher scores reported in users.	Favoring LTOT: significantly improved performance reported in users	Favoring LTOT
Chen 2023	Not assessed	Favoring LTOT: significantly higher scores in users with 86% reduction in impairment risk for users in rural areas	Not assessed	Favoring LTOT
Negro 2015	Neutral: no significant difference between two groups	Not assessed	Favoring LTOT: significantly better performance on TMT‐A and TMT‐B among users.	Favoring LTOT/Neutral
Çilingir 2020	Negative effect: scores were lower in LTOT users	Not assessed	Not assessed	Against LTOT

## 4. Discussion

This systematic review synthesized the available evidence on the impact of LTOT on cognitive function in patients with COPD from five studies. Overall, the findings suggested that LTOT may confer some cognitive benefits to patients, especially in domains related to global cognition and executive functions. Even though not all studies reported consistent benefits of LTOT on cognition, most of the studies included in our review showed a favorable association between LTOT use and cognitive performance. Only Cilingir et al. [19] reported worse cognitive outcomes in LTOT users as compared with nonusers. This could be attributed to lower FEV1% and higher baseline hypoxemia among the LTOT group in their study. Hence, worse cognitive outcomes in this group may reflect disease severity rather than a detrimental effect of LTOT itself.

Across the included studies, cognitive impairment was common among patients with COPD, with reported prevalence ranging from approximately one‐third to more than half of patients, depending on the cognitive tool used. Studies employing comprehensive or executive‐function–sensitive instruments such as the MoCA and TMT consistently identified a higher burden of cognitive impairment compared with those using MMSE. A meta‐analysis demonstrated that MoCA yields a greater sensitivity of up to 80.5% and specificity of 81.2% for detecting mild cognitive impairment compared with MMSE with sensitivity and specificity of 66.3% and 72.9%, respectively [22]. Likewise, another study in the elderly population reported that MoCA had higher sensitivity and specificity than MMSE for cognitive impairment [23]. This difference in the measurement of cognitive impairment is particularly salient in COPD, where the cognitive phenotype is characterized by “frontal‐executive” dysfunction rather than primary amnestic deficits. MMSE is significantly weighted toward language, basic memory, and orientation, which are usually preserved in stable COPD. The MoCA includes executive challenges such as the Clock Drawing Test and other activities that test for attention, abstraction, and visuospatial skills [24, 25]. Furthermore, the accuracy of cognitive screening tests like MMSE can be greatly affected by language and cultural differences of the study population. For example, Çilingir et al. [19] concluded that a high burden of cognitive impairment among their participants might have been partly due to many elderly participants having low literacy and family members informally translating test instructions. The MMSE is also susceptible to a “ceiling effect,” potentially overlooking mild cognitive impairment in patients with a higher educational level. This could be observed in the findings of Karamanli et al. [18], when LTOT users displayed normal global scores on the MMSE while having significantly lower scores on the MoCA. Timed instruments like the TMT‐A and TMT‐B give a more granular measure of processing speed. Dal Negro et al. [20] found that processing speed and multitasking domains assessed by TMT are the ones that improve most when patients strictly adhere to the LTOT protocol. Thus, MoCA and TMT may be better suited to evaluate cognitive impairment in COPD populations.

We also observed that clinical heterogeneity among studies further contributed to inconsistent findings. First, differences in COPD severity and baseline hypoxemia likely influence the potential benefit derived from LTOT. Even though almost all participants in the included studies were hypoxemic in the absence of exacerbation, the levels of hypoxia varied among the patients. While Dal Negro et al. [20] and Karamanli et al. [18] focused exclusively on patients with severe hypoxia (GOLD Stage 4), studies like Chen et al. [21] and Thakur et al. [8] studied participants spanning GOLD Stages 1 through 4. Importantly, LTOT users were the participants with more advanced disease and more profound hypoxemia. Second, the duration of LTOT exposure varied widely, ranging from months to several years. This is important to note because the short‐term use of LTOT may be insufficient to produce measurable cognitive benefits, particularly if structural or microvascular brain changes have already occurred [7]. Additionally, the prescribed daily duration of LTOT also differed among the studies. Third, adherence to prescribed oxygen therapy was rarely quantified among studies, which raises the possibility that reported LTOT exposure may not accurately reflect effective oxygenation in LTOT users. Hence, the differences in adherence among participants of multiple studies could have contributed to the heterogeneity among the studies. Apart from these treatment‐related factors, socioeconomic characteristics of the study population may further modify the relationship between LTOT and cognition. Chen et al. [21] reported differences in cognitive outcomes among rural and urban populations. The authors have suggested that access to healthcare resources, nutritional status, and comorbidities may influence both LTOT utilization and cognitive trajectories. However, there is a paucity of literature exploring the relationship between access to healthcare resources and its direct effect on LTOT use, adherence, and cognitive performance of COPD patients. Therefore, it is essential to interpret LTOT‐related cognitive outcomes within broader clinical and social contexts rather than attributing effects solely to oxygen supplementation.

The magnitude and consistency of the effects of LTOT on cognitive outcomes remained variable throughout the studies. The management of confounding variables in the included studies mostly depended on multivariate statistical modeling or stringent cohort matching. Thakur et al. [8] and Chen et al. [21] employed multivariate logistic regression to account for sociodemographic variables, such as age, educational level, and smoking status. Meanwhile, they controlled for COPD severity through FEV1% values to discern the distinct effect of oxygen therapy simultaneously. Dal Negro et al. [20] addressed potential bias by creating a well‐matched design, ensuring that the LTOT group and “as‐needed” cohorts were equivalent in baseline arterial blood gas values and age. Other studies, like Karamanli et al. [18], reduced confounding by using strict exclusion criteria to exclude participants with comorbidities, like cardiovascular diseases, that could independently cause cognitive impairment in these patients. Most studies took into account demographic and some primary physical confounders, but the inconsistent adjustment for socioeconomic factors such as occupation and educational level is still an important source of methodological heterogeneity.

A wealth of studies has proposed several mechanisms plausibly linking LTOT to improved cognitive function in COPD. Chronic hypoxemia has been associated with cerebral hypoperfusion, oxidative stress, and neuronal damage [26]. LTOT may ameliorate these processes by enhancing cerebral blood flow, which can reduce or prevent hypoxia‐induced neuronal injury [27]. The maintenance of cerebral blood flow despite increased arterial oxygenation, as observed by Hoiland et al. [13], pointed out that LTOT improves oxygen delivery to tissues in the state of chronic hypoxia in COPD. Our narrative synthesis demonstrated that the neuroprotective benefit of LTOT is most pronounced in population experiencing severe baseline hypoxemia (Pa02 < 55 mmHg). For instance, Dal Negro et al. [20] and Thakur et al. [8] studied participants with a mean age of 67–70 years and higher disease severity (FEV1% = 37%–52%), which is the age group where cerebrovascular insensitivity is most common. This shows that the stabilizing impact of LTOT on cerebral oxygen delivery is not universally assured but rather depends on the severity of the underlying disease. Additionally, LTOT may indirectly support cognition by improving exercise tolerance, sleep quality, mood, and overall functional status of patients [28]. Likewise, the selective improvement observed in executive and attentional domains of cognition across several studies is also intriguing. Executive functions rely heavily on frontal–subcortical circuits, which are particularly vulnerable to hypoxemic injury. Hence, restoration of oxygen delivery to these specific areas of the brain may provide measurable benefits on executive function performance, even when global cognitive scores remain unchanged [7, 29].

Our study has several limitations. First, all included studies were observational in design, which limits the causal inference of the effect of LTOT on cognition. Second, confounding factors such as disease severity, comorbidities, socioeconomic status, and education level were variably controlled across studies, producing heterogeneous results. Even though some studies reported adjusted analyses, the presence of residual confounding cannot be ruled out. Third, adherence to LTOT was rarely quantified among included studies, which made it difficult to assess dose–response relationships.

## 5. Conclusion

LTOT might have beneficial effects on the cognitive performance in patients with COPD. Further studies, including randomized controlled trials and longitudinal cohorts, are needed to establish causality and optimize LTOT treatment protocols for COPD.

Clinicians should consider cognitive screening as part of COPD management and promote adherence to LTOT wherever indicated.

## Funding

No funding was received for this manuscript.

## Ethics Statement

Ethical approval was not required for the systematic review.

## Consent

The authors have nothing to report.

## Conflicts of Interest

The authors declare no conflicts of interest.

## Supporting information


**Supporting Information** Additional supporting information can be found online in the Supporting Information section. PRISMA 2020 checklist. Table S1: Search strategy used in the current systematic review. Table S2: Quality assessment of included studies.

## Data Availability

The data that support the findings of this study are available from the corresponding author upon reasonable request.
